# Insecticidal Activity and Synergistic Combinations of Ten Different Bt Toxins against *Mythimna separata* (Walker)

**DOI:** 10.3390/toxins10110454

**Published:** 2018-11-04

**Authors:** Jing Yang, Yudong Quan, Prabu Sivaprasath, Muhammad Zeeshan Shabbir, Zhenying Wang, Juan Ferré, Kanglai He

**Affiliations:** 1 State Key Laboratory for Biology of Plant Diseases and Insect Pests, Institute of Plant Protection, Chinese Academy of Agricultural Sciences, No. 2, West Yuanmingyuan Road, Beijing 100193, China; cutejingyang@163.com (J.Y.); sivaprasathibt@gmail.com (P.S.); shann2347@yahoo.com (M.Z.S.); zywang@ippcaas.cn (Z.W.); 2 ERI de Biotecnología y Biomedicina (BIOTECMED), Department of Genetics, Universitat de València, 46100 Burjassot, Spain; yudongquan@126.com (Y.Q.); juan.ferre@uv.es (J.F.)

**Keywords:** *Bacillus thuringiensis*, Cry protein, Vip3 protein, oriental armyworm

## Abstract

The oriental armyworm (OAW), *Mythimna separata* (Walker), is a destructive pest of agricultural crops in Asia and Australia. Commercialized Bt crops have performed very well against their target pests; however, very few studies have been done on the susceptibility of OAW to Bt toxins in either sprays or expressed in Bt crops. In this work, we evaluated the toxicities of Cry1Ab, Cry1Ac, Cry1Ah, Cry1Fa, Cry2Aa, Cry2Ab, Cry1Ie, Vip3Aa19, Vip3Aa16, and Vip3Ca against OAW neonate larvae, as well as the interaction between Cry and Vip toxins. The results from bioassays revealed that LC_50_ (lethal concentration for 50% mortality) values ranged from 1.6 to 78.6 μg/g (toxin/diet) for those toxins. Among them, Vip3 proteins, along with Cry1A proteins and Cry2Aa, were the ones with the highest potency, with LC_50_ values ranging from 1.6 to 7.4 μg/g. Synergism between Cry and Vip toxins was observed, being high in the combination of Vip3Aa16 with Cry1 toxins, with synergetic factors ranging from 2.2 to 9.2. The Vip3Ca toxin did not show any synergistic effect with any of the toxins tested. These results can help in designing new combinations of pyramiding genes in Bt crops, as well as in recombinant bacteria, for the control of OAW as well as for resistance management programs.

## 1. Introduction

*Bacillus thuringiensis* (Bt) is a gram-positive bacterium that produces pesticidal crystal proteins which are active against insects, nematodes and other invertebrates [1,2,3]. Since Hannay discovered the crystalline inclusion in sporulating cells of Bt [4], the crystal proteins have been studied extensively to control the lepidopteran pest [1,5,6]. Because of the lack of toxicity to vertebrates and most other nontarget organisms, Bt toxins are environmentally friendly, either applied as biopesticides, or in transgenic crops [1,5,6]. Currently, transgenic crops producing Bt toxins (Bt crops) that kill major target pests have been globally adopted and are grown in more than 70 million hectares annually since 2010, having reached over 100 million hectares in 2017 [7]. Despite the high number of Bt toxins discovered to date, only a few Bt toxins, such as Cry1Ab, Cry1Ac, Cry1F, Cry2Ab, Cry3, Cry1C, and Vip3A, are found in commercial Bt crops [8]. Although they show a high efficacy against some pests, each one of these toxins has its own insecticidal spectrum of toxicity and, therefore, only a few of them are appropriate to control a given pest [9,10,11,12,13,14]. The evolution of insect resistance is the primary threat to the continuous success of Bt crops [15,16]. To deal with the above two issues, strategies of stacking (combining toxins that have different insect targets) and/or pyramiding (combining toxins that have the same insect target) two or more insecticidal traits in Bt plants have been deployed, with excellent results [7]. These strategies have been shown to be very effective in achieving a broader spectrum for insect control and combating resistance [17,18,19,20,21,22,23]. Therefore, the characterization of which set of Bt toxins is useful in the control of a given pest is of paramount importance to predict the efficacy of Bt crops on an insect pest that has not been tested previously, and even to predict the chances of developing resistance based on previous information on cross-resistance episodes in other related species.

Both Cry and Vip3 toxins follow similar steps in their mode of action. They are synthesized as protoxins, which must be cleaved by midgut proteases to be converted into active toxins after intake by the larvae [24,25]. Activated toxins insert into the epithelial cell membrane via binding to the specific brush border receptors and elicit the formation of pores, which eventually lead to the destruction of midgut epithelial cells [26,27,28,29,30,31]. These events ultimately lead to the death of larvae by septicemia [32,33]. Despite Cry and Vip3 toxins following similar steps in the mode of action, they differ in that they do not compete for binding sites [34,35,36,37,38]. A combination of Vip3Aa/Cry1Ac in cotton, Vip3Aa/Cry1Ab in corn, Vip3Aa/Cry1Ac/Cry1Fa in cotton, and Vip3Aa/ Cry1Ab/Cry1Fa in corn have been applied for combating resistance to the first-generation Bt cotton and corn that express Cry1Ac and Cry1Ab toxins, respectively [17,19,20,39]. The distinct mode of action between Cry and Vip3 proteins makes them good candidates to be combined in Bt crops to delay resistance evolution and broaden the insecticidal spectrum of the crop.

The oriental armyworm (OAW), *Mythimna separata* (Walker), (Lepidoptera: Noctuidae), is a well-known long-distance migratory and major agricultural pest in Asia and Australia [40,41,42,43,44,45,46,47]. Practically, control of OAW relies on chemical insecticide spray [48,49]. The adoption of Bt maize would provide new and alternative tools for limiting the damage caused by this pest. A laboratory study showed that *Mythimna unipuncta* could evolve resistance to Cry1Ab [50]. In addition, the fall armyworm (FAW), *Spodoptera frugiperda* (another species of the same family and a long-distance migratory moth in America), which also can feed on maize [51], has been reported to have developed resistance to Cry1Fa in Bt maize [52,53,54]. Therefore, research on the efficacy and interaction effects of Bt toxins on OAW are urgent and needed in ecological safety, as well as in building a biological control method for this pest.

In this work, we evaluated the efficacy of individual Cry (Cry1Ab, Cry1Ac, Cry1Ah, Cry1Fa, Cry1Ie, Cry2Aa, and Cry2Ab) and Vip3 proteins (Vip3Aa19, Vip3A16, and Vip3Ca), with an interest in maize crop protection. In addition, we studied the possible synergistic effects of combinations of Cry and Vip3 proteins.

## 2. Results

### 2.1. Susceptibility of Mythimna separata to Bt Toxins

The toxicities of different Bt toxins against OAW varied considerably (Table 1). The LC_50_ values ranged from 1.6 to 78.6 μg/g. Vip3Aa19 was the toxin with the lowest LC_50_ value (1.6 μg/g), though not significantly different from Vip3Ca and Cry1Ac. However, considering the LC_50_ and LC_95_ values together, Vip3Aa19 and Vip3Ca were the most potent toxins, followed by Cry1Ab, Cry1Ac, Cry2Aa, and Vip3Aa16. The rest of the toxins tested (Cry1Ah, Cry1Fa, Cry1Ie, and Cry2Ab) had significantly less potency, either because of having high LC_50_ values or by having a low regression slope. Cry1Ie was significantly the less toxic protein, with an LC_50_ value of 78.6 μg/g.

### 2.2. Effect of Bt Toxins Combinations against Mythimna separata

In the search for possible synergistic interactions between Cry and Vip3 toxins, different toxin combinations were tested against the OAW neonates and results are shown in Table 2. There were four combinations showing a statistically significant interaction, all of them with Vip3Aa16 (marked with an asterisk in Table 2). For these synergistic interactions, the synergistic factor (*SF)* ranged from 2.2 to 9.2. No significant antagonistic effects were found.

### 2.3. Determination of the Mortality Rate

The interaction of some combination of toxins was tested by the mortality rate at a fixed toxin concentration instead of the LC_50_ values. Of the four combinations tested, only the Cry2Aa/Vip3Ca combination showed a significant result (synergism), with an observed mortality of 69.8% vs. the expected 50% (Table 3).

## 3. Discussion

It is known that insects have varying degrees of susceptibility to different Bt toxins, and the assessment is necessary for defining susceptibility before the implementation of commercial cultivation of Bt crops. In this study, the toxicity (LC_50_) and synergistic effects of Cry and Vip3 toxins were assessed against OAW. The LC_50_ values obtained for OAW ranged from 1.6 to 78.6 μg/g for Cry and Vip toxins (Table 1). In previous reports, FAW and *Ostrinia nubilalis* showed higher susceptibility to Cry1Fa [54], which is even more effective than Cry1Ab and Cry1Ac against *Helicoverpa armigera* [55] or *Plutella xylostella* [56]. Co-expressing Cry1F with Cry1Ac in cotton, and Cry1Ab in maize, can broaden the number of targets species of Bt cotton and Bt maize [39,57]. However, in this study, Cry1Fa was less efficient than Cry1Ac and Cry1Ab. This indicates differences in susceptibility to those toxins between OAW and FAW. This suggests that pyramiding Cry1F and Cry1Ab in maize may not increase the efficacy against OAW control.

The LC_50_ and LC_95_ values of Vip3Aa19 and Vip3Ca did not show any significant difference. However, the slope showed a significant difference (Table 1), thus indicating that the toxicity of Vip3Ca was greater than that of Vip3Aa19. Although the value of Vip3Ca was not significantly different from those of Cry1Ab, Cry1Ac, and Cry2Aa at the LC_50_ level, it was significantly more active at the level of LC_95_. Vip3Ca showed remarkable efficacy against the OAW, according to both the LC_50_ and LC_95_ values, which clearly indicates that Vip3Ca could be recommended in controlling the OAW. Similarly, high slope values of Vip3A proteins were also reported in *Heliothis virescens*, which can be interpreted as that the activity of Vip3A proteins need a particular threshold concentration to be toxic in the insect midgut [58]. In a recent study, it was reported that Vip3Ca was as effective as Cry1Ab against *Ostrinia furnacalis*, but Vip3Aa was less toxic [59]. In addition, Vip3Ca was also found to overcome resistance to Cry1Ab in Cry1Ab-resistant *O. furnacalis*, indicating that Cry1Ab and Vip3Ca may have different binding sites. This suggests that co-expressing Cry1Ab and Vip3Ca may be a useful component in OAW and *O. furnacalis* pest management programs, as well as pest resistance management programs.

The interaction effect between proteins is an important issue in the selection of toxins to use in pest control and insect resistance management. A number of investigations have confirmed that synergism and antagonism may occur between Vip3 and Cry proteins [57,58,59,60]. Bergamasco et al. reported the species specificity effect of protein interaction between Cry1Ia and Vip3Aa [61,62,63]. A synergistic effect was found in FAW, *Spodoptera albula*, and *Spodoptera cosmioides*, but an antagonistic effect was found in *Spodoptera eridania*. In the present study, the highest synergistic effect was observed in the combination of Cry1Ie and Vip3Aa16. Cry1Ie is highly toxic to *O. furnacalis* [64] and effective in preventing the development of resistance in *H. armigera* targeted by Bt maize [65]. This suggests the potential use of combination of Cry1Ie and Vip3Aa16 in maize for a broader target species control and favoring the pest resistance management.

Cry1F expressing maize TC1507 is toxic to *O. nubilalis* [66], a relative species to *O. furnacalis*. Cry1F expressing maize has the potential to control *O. furnacalis*, as evidenced from results from both laboratory studies with pure protein [67] and field trials with TC1507 maize (unpublished data). In the present study, we found Cry1Fa and Vip3Aa16 interacted synergistically, with an *SF* of 6.3. This indicates that a pyramided maize plant expressing Cry1Fa and Vip3Aa16 would be a new strategy in an overall pest management program for both *O. furnacalis* and OAW. Meanwhile, synergistic effects were also observed in the combinations of Vip3Aa16 with either Cry1Ab (2.2-fold) or Cry1Ah (2.5-fold), although they were relatively low. Interestingly, we did not find any synergistic effect in the combinations of Vip3Ca with Cry1 type toxins (Table 2 and Table 3), although previous studies suggested that the mode of action of Vip3Aa was similar to that of Vip3Ca [31,68,69]; the difference in synergistic properties may unravel differences in their mode of action at the biochemical level.

## 4. Conclusions

This study demonstrated the toxicity of seven Cry and three Vip3 protoxins to OAW. Our results reveal that, among the Bt toxins tested, Vip3Aa19 and Vip3Ca have the highest toxicity, followed by Cry1Ab, Cry1Ac, Cry2Aa, and Vip3Aa16, whereas Cry1Fa, Cry1Ah, Cry2Ab, and Cry1Ie possess the lowest larvicidal activity. Combinations of Vip3Aa16 with Cry1 toxins as pyramids showed a significant synergistic activity, while combinations of Vip3Ca with Cry1 toxins did not show any synergism. The results obtained provide precise information for projecting new combinations of Bt genes in transgenic crops for a broader target spectrum and a reliable component of pest resistance management programs.

## 5. Materials and Methods

### 5.1. Insect Strains

Eggs of OAW were obtained from Keyun Biology Company. The eggs were transferred to Zip-lock bags (#10) and reared under laboratory conditions (28 ± 1 °C, L:D = 16:8 h, RH = 70%~80%). Neonates were used for diet bioassay within 6 h after hatching.

### 5.2. Diet Formulation

Artificial diet was formulated according to Wang and Zheng [70] with slight modifications. Ingredients: 250 g of powdered rat feed, 300 g of powdered corn leaf, soybean flour 40 g, corn flour 50 g, wheat germ 30 g, casein 20 g, yeast 40 g, glucose 20 g, fructose 20 g, sugar 30 g, Weber’s salt mixture 10 g, ascorbic acid 6 g, cholesterol 2 g, mixed vitamins 1 g, sorbic acid 6 g, erythromycin 1.2 g, and thiabendazole 2 g. These powders were mixed together, vacuum packed, and stored at 4 °C until use.

### 5.3. Bt Toxins

Trypsin-activated Cry1Ab, Cry1Ac, Cry2Aa, Cry2Ab, and Cry1Fa toxins were purchased from Envirologix (Portland, OR, USA). Cry1Ie and Cry1Ah are chromatographically purified recombinant proteins expressed in *Escherichia coli* and *Bacillus thuringiensis* (Biot 1 Ah), which were provided by the Chinese Academy of Agricultural Sciences, Biotech group. Vip3Aa16 and Vip3Ca were provided by the Department of Genetics, University of Valencia (Valencia, Spain). Vip3Aa19 was provided by the Da Bei Nong Group.

### 5.4. Bioassays

#### 5.4.1. Bt Toxin Bioassay

Ten toxins were used to check their toxicity against OAW neonates. Initially, the Bt toxins stock solutions (1 mg/mL) were prepared by dissolving the toxins in sodium carbonate buffer (50 mM, pH = 10) separately in their respective vials. In a 50 mL beaker, the protoxins were mixed thoroughly with the diet (3.5 g) and distilled water (6.5 mL). The Bt toxicity screening was performed by increasing the concentration gradually according to Shabbir et al., 2018 [71]. This mixture was divided equally into the cells of a 48-well plate. One OAW neonate was placed on the surface of the diet in each well, using a fine brush. These plates were sealed with sealing film and a small hole was punctured on each well and these plates were placed in the rearing room (28 ± 1 °C, L:D = 16:8 h, RH = 70%~80%). The survival rate and larval weight were recorded after 7 days. Larva weighed <0.2 mg and was beyond second instar, so it was considered dead.

#### 5.4.2. Assessment of Synergism between Bt Toxins

The synergetic effect between Bt toxins was assessed using combinations of the Cry and Vip3 families. In choosing the toxin ratios, the Cry1Ab is exemplified in order to bring the expected LC_50_ values of the toxin mixture to an appropriate range. A ratio of 0.7/0.3, or close to it, was chosen for ease of comparison and calculation. The diet bioassays for different combinations of Bt toxins were carried out with the same method as the one mentioned above for single toxins. Three replications were performed for each combination.

### 5.5. Statistical Analysis

PoloPlus (v 1.0, LeOra Software, Parma, MO, USA) was employed to estimate the 50% lethal concentration (LC_50_) with 95% fiducial limits (FL) and the slope for bioassays by probit analysis. Tests for expected and observed mortalities were evaluated using the method described by Tabashnik [68]. Differences between observed and expected LC_50_ values were analyzed by the U-test, that is, the LC_50_ ratio test [72]. The difference between the observed and expected mortalities was analyzed by conducting a χ^2^-test. Both U-test and χ^2^-test were run through the SAS 9.4 software (North Carolina State University, Raleigh, NC, USA).

## Figures and Tables

**Table 1:** Effect of Cry and Vip3 family protoxins against *Mythimna separata* neonates.

Toxin	*n*	LC_50_ (95%FL) μg/g *	LC_95_ (95%FL) μg/g *	Slope ± SE *	χ^2^	*df* (χ^2^)
Cry1Ab	480	6.4 (4.2, 9.0) c	326.5 (158.3, 1034.2) a	0.96 ± 0.12 bc	3.3	5
Cry1Ac	480	3.7 (2.1, 5.7) cd	255.0 (121.0, 893.7) a	0.89 ± 0.12 bc	1.6	5
Cry2Aa	528	6.2 (3.9, 9.8) c	725.6 (229.7, 5792.6) a	0.80 ± 0.12 c	4.2	6
Cry2Ab	768	22.3 (15.3, 32.3) b	>1000	0.82 ± 0.06 c	20.8	14
Cry1Fa	672	14.4 (5.7, 24.6) b	>1000	0.22 ± 0.04 c	12.8	10
Cry1Ie	672	78.6 (47.3, 160.3) a	>1000	0.86 ± 0.11 c	13.7	9
Cry1Ah	576	18.7 (13.1, 25.9) b	>1000	0.26 ± 0.03 c	9.9	8
Vip3Aa16	528	7.4 (2.7, 19.3) c	351.8 (74.4, 939.9) a	0.98 ± 0.14 bc	17.5	6
Vip3Aa19	384	1.6 (0.55, 3.53) d	35.0 (17.0, 148.0) b	1.24 ± 0.15 b	11.2	6
Vip3Ca	480	3.4 (2.5, 4.6) cd	27.1 (17.4, 54.9) b	1.83 ± 0.19 a	5.2	5

*n*, Number of larvae tested. 95%FL, 95% fiducial limits. *, Values followed by the same lowercase letter in the same column indicate no significant difference at *p* ≥ 0.05. SE, Standard error.

**Table 2:** Susceptibility of *Mythimna* *separata* neonate larvae to combinations of Cry and Vip protoxins.

Toxins	Ratio	*n*	Slope ± SE	LC_50_ (95%FL) μg/g		χ^2^	*df * (χ^2^)	*SF*	*p*
				Observed	Expected				
Cry1Ab/Vip3Aa16	0.71:0.29	480	0.9 ± 0.1	3.1 (1.8, 4.5)	6.6 (3.6, 10.7)	2.8	5	2.2	0.03
Cry1Ab/Vip3Ca	0.71:0.29	480	1.0 ± 0.1	2.6 (0.4, 5.5)	5.1 (3.5, 7.1)	15.1	5	2.0	0.29
Cry1Fa/Vip3Aa16	0.71:0.29	480	1.0 ± 0.1	1.8 (0.3, 3.9)	11.3 (4.3, 22.8)	9.1	5	6.3	0.01
Cry1Fa/Vip3Ca	0.71:0.29	480	1.2 ± 0.1	11.7 (5.7, 21.8)	7.5 (4.1, 10.9)	11.3	5	0.6	0.29
Cry1Ie/Vip3Aa16	0.67:0.33	480	0.6 ± 0.1	2.0 (0.57, 3.9)	18.6 (7.2, 46.7)	1.3	5	9.2	<0.01
Cry1Ie/Vip3Ca	0.67:0.33	480	0.9 ± 0.1	6.20 (1.4, 13.9)	9.5 (6.7, 12.9)	13.6	5	1.5	0.48
Cry1Ah/Vip3Aa16	0.68:0.32	480	1.5 ± 0.1	5.12 (3.4, 7.1)	12.6 (5.9, 23.4)	1.7	5	2.5	0.02
Cry1Ah/Vip3Ca	0.68:0.32	480	1.6 ± 0.2	8.12 (5.7, 10.7)	7.8 (5.5, 10.4)	0.9	4	1.0	0.84
Cry2Aa/Vip3Ca	0.50:0.50	480	1.4 ± 0.2	4.07 (2.4, 6.2)	4.4 (3.0, 6.2)	7.8	5	1.1	0.78

*n*, Number of larvae tested. SE, Standard error. 95%FL, 95% fiducial limits. *SF*, Calculated as the expected LC_50_ divided by the observed LC_50_. *p*, Probability value based on U-test.

**Table 3:** Mortality of *Mythimna* *separata* neonate larvae to combinations of Cry and Vip protoxins.

Toxins	Proportion	Concentration (μg/g)	*n*	Mortality ± SE (%)		*p*
				Observed	Expected	
Cry1Ac/Vip3Aa16	0.56/0.44	2.5	144	55.6 ± 0.9	50	0.69
Cry1Ac/Vip3Ca	0.56/0.44	1.8	144	48.3 ± 0.2	50	0.83
Cry2Aa/Vip3Aa16	0.50/0.50	3.4	144	57.8 ± 0.1	50	0.35
Cry2Aa/Vip3Ca	0.50/0.50	2.2	144	69.8 ± 0.3	50	0.02

*n*, Number of larvae tested. SE, Standard error. *p*, Probability value based on χ^2^-test.
